# Supplementary material for: Synthesis of Layered Double Hydroxides with Phosphate Tailings and Its Effect on Flame Retardancy of Epoxy Resin
Source: Polymers (Basel). 2022 Jun 21;14(13):2516. doi: 10.3390/polym14132516 (PMC9268921; doi:10.3390/polym14132516)
Supplement: Supplementary file 1 [file polymers-14-02516-s001.zip › polymers-1764925-supplementary.pdf]

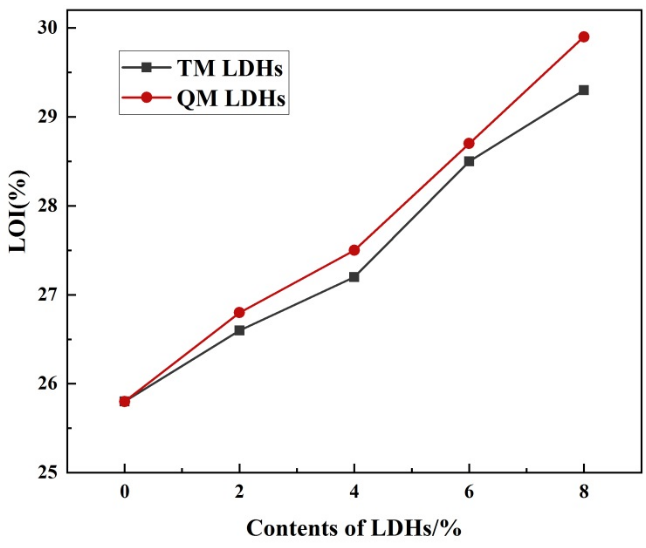

Figure S1. LOI trends curves of LDHs-1/EP and LDHs-2/EP composites.

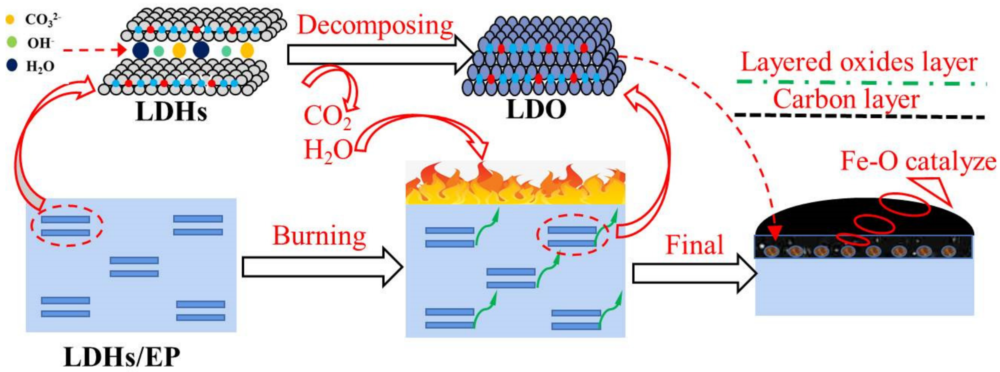

Figure S2. Possible flammability and charring process of EP composites.
